# The Effects of Cognitive–Motor Dual-Task Exercise and Exergaming on Balance and Functional Mobility in Children with Down Syndrome: A Comparative Randomized Trial

**DOI:** 10.3390/brainsci16010079

**Published:** 2026-01-06

**Authors:** Safia Darweesh Halwsh, Maha F. Algabbani, Samiah Alqabbani, Tahani AbdulAziz Alahmad, Muneera M. Almurdi, Reema A. Alshubaily, Mai Aldera, Hawra’a Abdullah Al-Dubisi, Ruaa Mohammed Almedlej, Afaf A. M. Shaheen

**Affiliations:** 1 Department of Rehabilitation Health Sciences, College of Applied Medical Sciences, King Saud University, Riyadh 11433, Saudi Arabiamaldera@ksu.edu.sa (M.A.); ashaheen@ksu.edu.sa (A.A.M.S.); 2 Department of Rehabilitation Sciences, College of Health and Rehabilitation Sciences, Princess Nourah Bint Abdulrahman University, Riyadh 84428, Saudi Arabia; sfalqabbani@pnu.edu.sa; 3 Department of Physical Therapy, Voice of Down Syndrome Association, Riyadh 11693, Saudi Arabia; 4 Basic Sciences Department, Faculty of Physical Therapy, Cairo University, Cairo 12613, Egypt

**Keywords:** down syndrome, balance, functional mobility, exergaming, dual-task, pediatric rehabilitation

## Abstract

**Background/Objectives:** Children with Down Syndrome (DS) exhibit difficulties in maintaining balance and coordination in addition to limitations in functional mobility. The Cognitive–Motor Dual-Task Exercise Program (CMDT) has shown the ability to improve balance and functional mobility. This study aimed to compare the effect of CMDT versus exergaming on the balance and functional mobility of children with Down Syndrome aged 8–14 years. **Methods:** A randomized comparative trial was conducted, dividing participants’ children with DS into two intervention groups: CMDT group and exergaming group. Participants were recruited using convenience sampling methods from the Voice of Down Syndrome Association and the Down Syndrome Charitable Association in Riyadh, Saudi Arabia. Both interventions were implemented over a period of six weeks. Outcome measures included the Timed Up and Go (TUG), Four Square Step Test (FSST), and Pediatric Balance Scale (PBS). **Results:** A total of 23 children with DS participated in the study. A mixed repeated measures ANCOVA shows a significant effect of time across the two groups (*p* < 0.001) for TUG, FSST, and PBS, indicating improvements in balance and functional mobility. There were no significant differences between the two group interventions. **Conclusions:** CMDT and exergaming were equally effective in improving balance and functional mobility in children with DS. **Trial Registration:** Clinicaltrials.gov with ID NCT06146907.

## 1. Introduction

Down Syndrome (DS) is a genetic condition caused by the presence of an extra chromosome 21. This additional genetic material leads to various physical characteristics and cognitive differences. It is estimated that DS affects approximately 1 in 1000 to 1 in 1100 live births [1].

Individuals with DS may face challenges in learning, speech, and motor skills, as well as certain health-related issues.

A notable characteristic in children with DS is delayed motor development compared to typically developing peers. Children with DS often exhibit lower motor performance and reduced cognitive abilities when compared to peers of the same age or mental age (i.e., the level of cognitive functioning typical for a specific age group) [2]. These challenges can result in difficulties in achieving balance, coordination, and functional mobility. Physical therapy interventions play a critical role in addressing these issues and improving overall outcomes.

Dual-task (DT) intervention is a rehabilitation method used in physical therapy that involves performing a primary task (such as walking) while simultaneously engaging in a secondary task (for example, talking or carrying an object). Both tasks are distinct and can be carried out independently, each with its own objective. This technique is designed to enhance cognitive and motor abilities by requiring the individual to divide their attention and multitask effectively [3]. As DT enables individuals to engage in motor tasks while simultaneously performing higher-level cognitive functions, it is an essential component of daily life [4].

Exergaming, which combines physical exercise with video games, functions as a DT training method where players perform motor exercises while simultaneously engaging in cognitive activities [5,6]. Exergaming is hypothesized to enhance functional mobility through several key theoretical mechanisms. Unlike traditional physical therapy, these platforms require enhanced attentional control to process simultaneous visual and auditory stimuli. Furthermore, the integration of immediate sensory feedback allows for real-time postural adjustments, which is critical for children with DS who often struggle with proprioceptive processing. Finally, the gamified nature of the intervention boosts intrinsic motivation, increasing the volume and intensity of repetitions necessary for neuroplastic changes and motor skill consolidation [7,8,9]. Through this interactive and engaging format, therapy or exercise can be made more enjoyable and engaging for children. Exergaming has been used as a therapy for children with DS [10,11,12,13].

A significant limitation of exergaming intervention is its dependence on specialized devices, which can be challenging to implement in all clinical settings or at home. This restricts the widespread access to this therapy. Consequently, there is a need for alternative dual-task programs that are more accessible and provide the benefits of combined physical and cognitive training without requiring specialized exergaming equipment.

In 2023, Büyükçelik et al. developed the Cognitive–Motor Dual-Task Exercise Program (CMDT) [14]. The CMDT consists of a variety of motor activities (such as walking, sitting, and jumping) that are performed once with motor tasks (such as carrying an empty box) and once with cognitive tasks (such as naming some fruits and vegetables). It can be implemented in various settings without specialized equipment. In addition, this program demonstrated an ability to improve the balance and functional mobility of DS children [14]. Despite the distinct advantages of exergaming and the clinical accessibility of CMDT, there is currently a lack of comparative evidence regarding their effectiveness for children with DS. This study aims to compare these two intervention modalities to determine which offers greater improvements in balance and functional mobility, providing evidence-based guidance for future pediatric rehabilitation.

Because both exergaming and CMDT interventions utilize dual-task principles by integrating cognitive processing with physical movement, we hypothesized that both protocols would yield comparable improvements in balance and functional mobility.

## 2. Materials and Methods

### 2.1. Study Design and Setting

Using convenience sampling, this randomized comparative trial was carried out from September to December 2024. Participants were recruited by phone call from the Voice of Down Syndrome Association and the Down Syndrome Charitable Association in Riyadh, Saudi Arabia.

### 2.2. Participants

The participants were children with DS (aged 8–14 years, both genders). Children with uncontrollable medical conditions or seizures, impairments that limited their activity, such as spinal deformities, or those who were uncooperative or unable to follow instructions were excluded. Furthermore, children who played video games daily(defined as ≥1 h per day), as indicated by their parents, were excluded to avoid the extraneous activity learning effect [15].

### 2.3. Randomization and Blinding

A randomized double-blinded design was utilized, wherein the children and their families were unaware of the intervention delivered to the other group, while the examiner was blinded to the group assignment. The examiner was only present during testing sessions and had no access to intervention records or treatment rooms. To ensure an objective assessment, the evaluator was instructed not to engage in conversation regarding the nature of the child’s treatment. While the interventions were physically different, families were informed that the study was comparing “two different but potentially beneficial therapeutic approaches.” They were not told which group was the “experimental” group versus the “active comparator” group. Families from the two groups attended different sessions at different times and in different rooms to prevent them from interacting and comparing the specifics of the interventions. Moreover, the statistical analysis was performed by a researcher who was blinded to group coding until the analysis was complete.

Participants were randomly assigned (using a 1:1 allocation ratio) to either the CMDT or exergaming groups via a computer-generated sequence. To balance the ages of the treatment groups, stratified randomization was used.

### 2.4. Sample Size

The sample size was determined using specialized software (Power Sample Size and Calculation Program, Version 3.1.6). To achieve a power of 0.90, a significance level of 0.05 was used (two-tailed), and to detect a large effect size (d = 0.8, based on Cohen’s conventions), 24 participants were needed, with 12 assigned to each group.

## 3. Outcome Measures

Assessments were performed at baseline and after the six-week intervention. Balance was measured using Pediatric Balance Scale (PBS) [16], and Functional Mobility using the Timed Up and Go Test (TUG) [17], and Four Square Step Test (FSST) [18,19].

### 3.1. Pediatric Balance Scale (PBS)

The Pediatric Balance Scale (PBS) is an adaptation of the adult Berg Balance Scale, designed specifically for children. It evaluates functional balance skills in school-aged children, typically between 5 and 15 years old, with mild to moderate motor or balance impairments [16]. The PBS has been validated for children with DS showing strong concurrent validity with Intelligence Quotient (IQ) scores (r = 0.738, *p* < 0.01) and high inter-rater reliability for overall test scores (ICC_3,1_ = 0.997) [16]. It includes 14 items representing everyday activities, such as sitting to standing, standing on one foot, turning, reaching, and retrieving objects from the floor. Each item is scored on a 0–4 scale, with 0 indicating the lowest function and 4 indicating independent task completion. The total score ranges from 0 point to 56 points, with higher scores reflecting better balance function. The test is administered in 15–20 min using basic equipment like a chair, stool, ruler, and stopwatch [16].

### 3.2. Timed Up and Go (TUG)

The TUG assesses balance, functional mobility, and walking ability, and has been validated for children and adolescents with DS [17,20], demonstrating concurrent validity. The TUG test had the strongest association with Dimension E (“Walking, Running, and Jumping”) of the Gross Motor Function Measure (GMFM), with a correlation value of r = −0.55 (*p* < 0.001) and also demonstrated good intrasession reliability, as shown by an intraclass correlation coefficient (ICC) of 0.82 [20]. The TUG test measures, in seconds, the time required for a participant to stand up from a chair, walk 3 m, turn around, walk back to the chair, and sit down again. The chair’s height utilized in the test permits the child’s hips and knees to flex at roughly 90° with their feet resting on the floor. The chair was with a backrest but no armrests [20]; these recommendations ensure that the chair is correctly sized for children, facilitating ease of standing and sitting during the test while not introducing additional difficulty due to poor chair height or design. Participants used the same chair each time they were tested to be able to compare scores. The test has been previously used with children with DS [21,22].

### 3.3. Four Square Step Test (FSST)

This assesses the dynamic balance and coordination of participants through evaluating their ability to step over objects in forward, sideways, and backward directions. The FSST has been validated for children with DS, showing concurrent validity with the TUG test (r = 0.74) and the Functional Reach Test (FRT), with Spearman’s ρ = −0.58 (95% CI: −0.86 to −0.13) [18].

During the test, participants were instructed to step over four sticks arranged in a “plus” shape on the floor as quickly as possible. Both feet of the participant must touch the floor in each square. The participant starts in Square 1 and follows these sequences: A clockwise path would be Square 1, Square 2, Square 3, Square 4, and then Square 1; a counterclockwise path would be from Square 4 to Square 3, then Square 2, then Square 1. The administrator demonstrates the test, and one practice trial is allowed to ensure understanding. Two trials were performed, and the better time (in seconds) is recorded as the score [18,23].

## 4. Interventions

**Figure 1:**
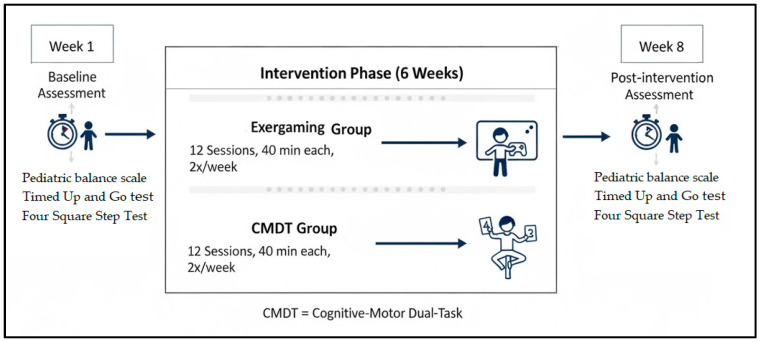
Schematic representation of the study procedure.

The whole study was conducted for a total of eight weeks. The first week was assigned to the baseline assessment of the participants. Intervention itself (either exergaming or CMDT) was conducted over a period of six weeks (from week two to week seven), involving 12 sessions of 40 min each. Finally, post-intervention assessments were completed in the eighth and final week of the study (Figure 1).

### 4.1. CMDT Group

Participants received the training program as it was presented in the study of Buyucelik et al., 2023 [14]. The CMDT intervention utilizes an adjustable chair height, without armrests, to ensure both feet touch the ground. It also included an empty box, a Pilates ball, and a soft exercise mat. Participants were provided with trial practice with each item [14]. The program includes five activities with a one-minute rest period following each activity:

1. Walking Tasks: Participants walked a 3 m distance on both hard and soft surfaces. This included walking while performing a motor task (carrying an empty box, 21 × 27 × 18 cm) for one minute on each surface, and walking while performing a cognitive task (naming fruits and vegetables) for one minute on each surface.
2. Seated Balance on a Pilates Ball: Participants sit on a Pilates ball with their feet on the floor. Tasks involved sitting for one minute with eyes open, then a one-minute eyes closed while performing a motor task (arms flexed to 90 degrees). Additionally, participants sat for one minute with their eyes open, then one minute with their eyes closed while performing a cognitive task (naming friends and relatives).
3. Two-Foot Forward Jumps: Participants performed two-foot forward jumps over a 3 m distance. This included jumping while carrying an empty box for two minutes, with 30 s interval rests. A subsequent two-minute period involved jumping while performing a cognitive task (naming colors in the room and the names of friends and family members), also with 30 s intervals of rest.
4. Single-Leg Standing: Participants stood on one leg (right then left) for 30 s while performing a motor task (elbow flexed to 90 degrees). After a one-minute rest, they repeated the single-leg stance for 30 s on each leg while performing a cognitive task (naming friends and relatives).
5. Sit-to-Stand Transitions: Participants completed 15 sit-to-stand repetitions, with a 5 s rest between each repetition, to achieve a total of 2 min of sit-to-stand activity from a chair while performing a motor task (carrying an empty box measuring 21 × 27 × 18 cm). This was followed by a 30 s rest interval, after which the same set of repetitions was performed while completing a cognitive task (naming fruits and vegetables).

### 4.2. Exergaming Group

The exergaming intervention employed a Nintendo Switch™ console equipped with a console unit and two Joy-Con controllers. The games were played on a 45-inch TV screen. The Nintendo Switch was set up with two controllers, one attached to the participant’s leg and the other held in their hand. Nintendo Switch™ Family—Nintendo—Official Site (https://www.nintendo.com/us/gaming-systems/switch/, accessed on 2 December 2025). Each of the five selected games was played for five minutes, with a one-minute rest between each game.

Five Family Trainer mini games were chosen based on insights from a previous pilot study [24]. These games were usable, engaging, enjoyable, and required the player to shift their weight and step while standing.

The selected mini games were as follows:

1. Sprint Challenge: Participants sprinted through the course by stepping in place as fast as they could to improve endurance.
2. Log Leaper: Players jumped over spinning logs with both legs, focusing on timing and rhythm to enhance coordination.
3. Timber Trail: This obstacle course required running, jumping, and dodging, designed to improve agility and coordination.
4. Jump Rope: Participants mimicked real jump rope motions; this game aimed to boost stamina and coordination through repetitive movements.
5. Head on Hurdler: A reaction-based game that increases in difficulty, requiring players to quickly dodge hurdles by jumping and stepping to improve speed and flexibility.

## 5. Statistical Analysis

The data were analyzed using IBM SPSS Statistics for Windows, version 29 (IBM Corp., Armonk, NY, USA). For continuous variables, the Shapiro–Wilk test was applied to determine the normality of the data. If the data followed a normal distribution, statistical analysis was reported as the mean and standard deviation. Otherwise, it was reported as the median and (1st–3rd quartiles). Categorical data were presented as frequency and percentage. An independent sample t-test for normally distributed data, the Mann–Whitney test for non-normally distributed data, and the Chi-square test for categorical data were used to compare the two groups at the baseline level.

The data were analyzed using mixed repeated measures Analysis of Covariance (ANCOVA) to investigate the effects of CMDT and Exergaming on Balance and Functional Mobility across two time points (baseline, post-intervention). Age and gender were selected a priori as covariates based on the established literature indicating that motor development trajectories and postural stability in children with DS are significantly influenced by biological maturation and sex-linked musculoskeletal differences [25]. Including these variables allowed us to isolate the specific effect of the intervention by controlling for these known sources of variance. All main effects and interaction effects from ANCOVA were interpreted after statistically adjusting for the effect of the covariates.

Statistical significance for all analyses was set at α = 0.05. The Shapiro–Wilk test indicates that all continuous variables data were normally distributed (*p* > 0.05), except for PBS (*p* < 0.5).

## 6. Results

### Participant Characteristics

**Figure 2:**
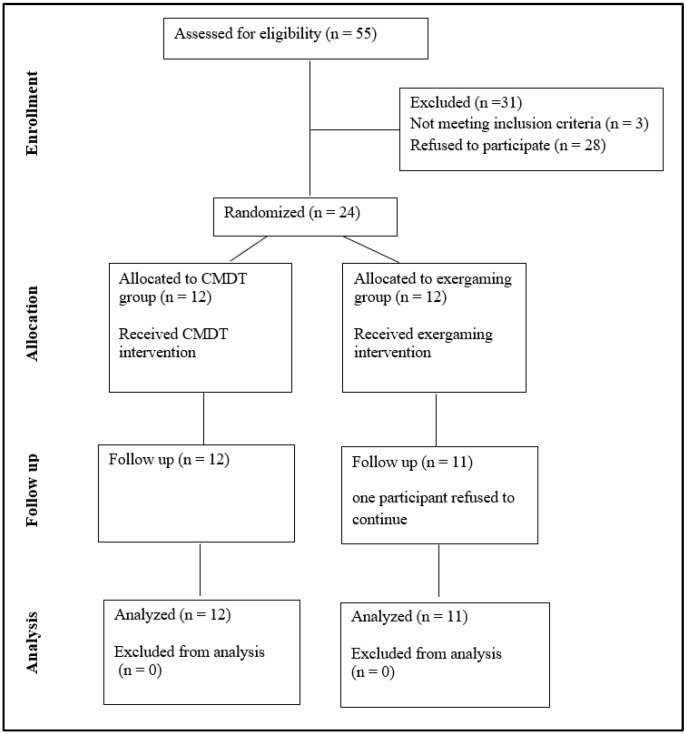
Flowchart diagram of participants.

Fifty-five children were contacted to participate. Only 27 children’s guardians signed the consent form and agreed to participate. Three were excluded as they cannot follow commands. A total of 24 participants, aged 8 to 14 years (12 males and 12 females), were eligible and included in the randomization process, then assigned to their respective groups. One child from the exergaming group was withdrawn from the intervention due to lack of interest in the games after only three sessions. A total of twenty-three participants completed the 6-week intervention: eleven in the exergaming group (10.45 ± 1.75 years, four males and seven females) and twelve in the CMDT group (11.50 ± 1.50 years, seven males and five females), and their data were analyzed. The participants’ flowchart diagram is shown in Figure 2.

Table 1 shows that participants’ characteristics and motor performance did not differ significantly between groups at baseline.

## 7. Effect of Covariates on the Outcome of Interventions

**Figure 3:**
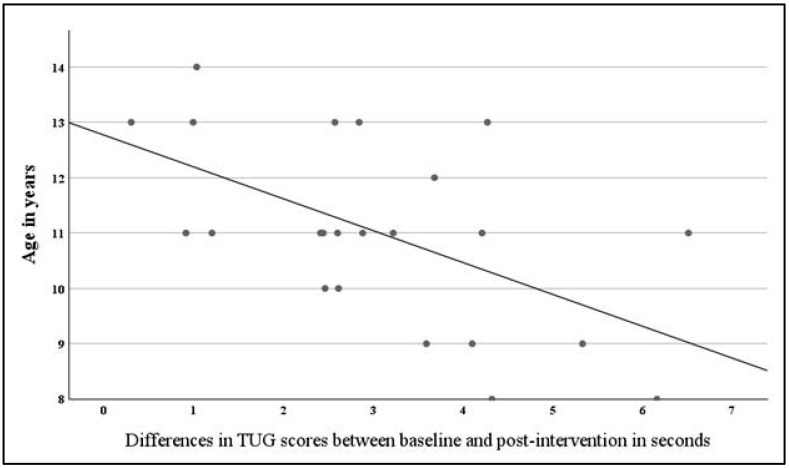
Age-related differences in treatment response based on ANCOVA: Younger participants demonstrate greater reductions in TUG scores compared to older participants.

The results of ANCOVA showed a main effect of age on TUG scores with large effect size (F_(1,18)_ = 6.99, *p* = 0.02, ηp^2^ = 0.28) and a significant interaction between time and age (F_(1,18)_ = 11.99, *p* = 0.003, ηp^2^ = 0.40), suggesting that the change in TUG over time varied depending on participants’ age. Figure 3 indicates that the reduction in TUG score differences between the baseline and post-intervention (reflecting improved functional mobility) were more pronounced in younger children.

**Figure 4:**
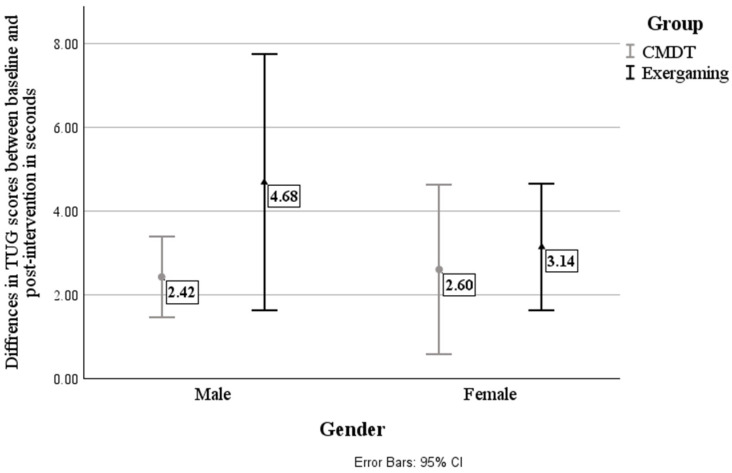
Interaction of gender and intervention type on TUG score improvements based on ANCOVA. Male participants demonstrated a more pronounced treatment response to exergaming compared to CMDT.

There was no main effect of gender on the TUG scores between subjects. However, a significant interaction between group and gender was also observed on TUG with large effect size (F_(1,18)_ = 11.384, *p* = 0.003, ηp^2^ = 0.66) as shown in Figure 4. Male participants in the exergaming group showed significantly greater improvement (mean change = 4.68, SD = 1.91) compared to males in the CMDT group (mean change = 2.42, SD = 1.04), indicating better performance with exergaming (*p* = 0.03). However, there was no significant difference in performance (*p* = 0.88) between female participants in the CMDT (mean change = 2.60, SD = 1.64) and exergaming (mean change = 3.14, SD = 1.63) groups.

In terms of FTTS and PBS scores, age and gender did not have significant main effects or interactions with time or groups.

### Intervention Effects

Both the CMDT group and the exergaming group demonstrated significant improvements in outcomes from their baseline measurements over time (F_(3,16)_ = 82.69, *p* < 0.001, ηp^2^ = 0.94). This indicates that both interventions were effective (Table 2). However, there was no significant difference observed between the groups (F_(3,16)_ = 0.06, *p* = 0.98, ηp^2^ = 0.01), suggesting comparable efficacy. Furthermore, no significant interaction between group and time was found (F_(3,16)_ = 0.02, *p* = 0.9, ηp^2^ = 0.004), reinforcing that neither intervention outperformed the other over the study period. This result confirms the hypothesis that there was no significant main effect of group type. This indicates that motor gains could be driven by the dual-task nature of both interventions.

## 8. Discussion

Our findings indicated significant improvements in balance and functional mobility over time for both CMDT and exergaming interventions, as reflected by improvements in TUG, FSST, and PBS scores with no significant differences between them.

The significant impact of age on TUG scores, along with a notable time-by-age interaction, indicates that younger children showed greater improvements in functional mobility. This aligns with research highlighting the critical period of neuroplasticity in early childhood, during which the brain is more adaptable to learning and change [26,27]. Young children with DS may have a more flexible neural system, enabling better integration of motor learning principles through the CMDT and exergaming, resulting in more significant mobility gains compared to older children. Early intervention is widely recognized as beneficial in developmental disorders, and these findings support its importance in enhancing motor skill development [28,29].

A significant interaction between group and gender on TUG scores was observed in the current study, suggesting that responses to the interventions varied by gender. Male participants in the exergaming group showed significantly greater improvements compared to males in the CMDT group. However, the reasons for this superior performance remain unclear as participant preferences were not recorded.

The current study found no significant difference in functional mobility improvement between female participants in the CMDT and exergaming groups, indicating that both interventions were equally effective for girls with DS. This suggests that the motivational or engagement factors that benefited males in the exergaming group were less pronounced for females, or that the CMDT was equally suited to their learning styles. In this study, girls may generally prefer activities involving balance, coordination, and rhythm; the specific exergames used in this study may not have resonated as strongly with female participants [30].

Although our methods differed in relation to the console used and the games included, our findings concur with previous research, revealing the positive effects of dual-task intervention [14], and the studies that investigate exergaming [10,31,32]. This suggests that exergaming is an effective intervention regardless of the platform chosen, while the present study emphasizes the Nintendo Switch as a modern platform approach. While Büyükçelik et al. (2023) [14] conducted an 8-week CMDT, the current study implemented 12 sessions over a shorter 6-week period. Despite this shorter timeframe, our participants showed significant improvements in all measured outcomes from baseline to post-intervention [14]. This finding is confirmed in another study that suggested that balance training exercise can yield an immediate effect after one week, a mid-term effect after three weeks, and a late effect after six weeks in healthy children aged 9 to 11 years of both genders [33].

The improvement in balance and functional mobility observed in this study can be attributed to several factors. Motor learning was enhanced as both interventions required participants to perform complex motor tasks, which likely improved their motor learning and control. This aligns with the findings of Wuang et al. (2011), who observed improvements in motor ability following a Wii-based intervention in children with DS [34]. Consequently, the cognitive components of both interventions may have facilitated enhanced motor abilities by improving attention and decision-making during movement. This confirms the suggestion that cognitive–motor correlation is crucial for balance and mobility, as proposed in previous studies [35]. The video game-based element of exergaming and the variety of activities in CMDT contributed to increased participant motivation and engagement, leading to greater continuous effort and practice [36,37]. The improvements in motor functions suggest that both interventions were effective in addressing the unique motor challenges faced by children with DS. The absence of significant differences between interventions suggests that clinicians and families can choose either approach based on their preferences, accessibility, and engagement.

## 9. Strengths and Limitations

Several limitations must be acknowledged, as they may affect the generalizability of the findings. The relatively small sample size may have reduced statistical power to detect differences between intervention groups. Conducting the study in a single-center could lead to a lack of diversity in the study population. Moreover, the study lacked long-term follow-up, preventing an assessment of the durability of the observed functional gains. Additionally, inherent variability in baseline motor and cognitive abilities among children with Down Syndrome may have influenced individual responses to interventions.

## 10. Conclusions

In conclusion, both CMDT and exergaming showed promising potential in enhancing balance and functional mobility in children with DS. While these findings suggest that such interventions could be valuable additions to clinical and home-based rehabilitation programs, the results should be interpreted with caution due to the study’s scope. Future research involving larger, multi-center trials is necessary to confirm these effects. Additionally, incorporating neurophysiological and cognitive metrics, along with long-term longitudinal follow-up, would provide deeper insights into how these functional gains are sustained.

## Figures and Tables

**Table 1:** Participants’ baseline characteristics.

Characteristics n	CMDT Group 12	Exergaming Group 11	Comparison at Baseline		
			Test	df	*p*
Age (years)	11.50 ± 1.50	10.45 ± 1.75	t = 1.54	21	0.14
Gender/boys	7 (58.3%)	4 (36.4%)	X^2^ = 1.11	1	0.29
Girls	5 (41.7%)	7 (63.7%)			
Height (cm)	133.92 ± 11.89	130.91 ± 10.18	t = 0.65	21	0.52
Weight (kg)	40.75 ± 13.82	34.45 ± 10.11	t = 1.23	21	0.23
BMI (kg/m^2^)	21.75 ± 5.64	19.36 ± 5.03	t = 1.06	21	0.30
TUG (s)	9.38 ± 2.38	10.16 ± 2.71	t = −0.73	21	0.47
FSST (s)	18.94 ± 5.63	19.55 ± 4.17	t = −0.30	21	0.77
PBS(points)	54.0 (52.5–54.0)	53.0 (52.0–54.0)	U = 44.50	21	0.17

n: number of participants, cm: centimeter, kg: kilograms, m: meter, s: seconds, BMI: body mass index, TUG: Timed Up and Go Test, FSST: Four Square Step Test, PBS: Pediatric Balance Scale. Data were expressed as the mean ± standard deviation, except for PBS, which is reported as the median and interquartile range (1st–3rd quartiles), and gender, which is presented as frequency and percentage. T = independent sample *t*-test. U = the Mann–Whitney test, X^2^ = chi-square test, df = degree of freedom. *p* significance < 0.05.

**Table 2:** Effect of time and intervention on TUG, FSST, and PBS scores using mixed repeated ANCOVA.

Outcome Measure		Baseline Mean ± SD n = 12	Post-Intervention Mean ± SD n = 11	Within Subject			Between Subjects		
				Sig	95% Confidence Interval		Sig	95% Confidence Interval	
					Lower Bound	Upper Bound		Lower Bound	Upper Bound
TUG (s)	CMDT	9.37 ± 2.38	6.88 ± 1.43	<0.01	2.71	3.80	0.73	−1.24	1.74
	Exergaming	10.15 ± 2.71	6.45 ± 1.54						
FSST (s)	CMDT	18.93 ± 5.36	14.58 ± 4.12	<0.01	2.44	5.69	0.97	−3.67	3.80
	Exergaming	19.55 ± 4.17	15.03 ± 2.91						
PBS (Points)	CMDT	53.33 ± 1.92	55.25 ± 0.87	<0.01	−3.02	−1.39	0.84	−0.94	1.14
	Exergaming	52.72 ± 1.48	55.27 ± 0.90						

Tests based on AVCOVA estimated marginal means. CMDT = Cognitive–Motor Dual-Task Exercise, n = number of participants, TUG: Timed Up and Go test, FSST: Four Square Step test, PBS: Pediatric Balance Scale. Sig = significance level, *p* < 0.05.

## Data Availability

The data presented in this study are available on request from the corresponding author due to participant’s privacy.
